# Hypoglycemic Effect of an Herbal Decoction (Modified Gangsimtang) in a Patient with Severe Type 2 Diabetes Mellitus Refusing Oral Anti-Diabetic Medication: A Case Report

**DOI:** 10.3390/medicina59111919

**Published:** 2023-10-30

**Authors:** Sungjun Joo, Hyonjun Chun, Jisu Lee, Seungmin Seo, Jungmin Lee, Jungtae Leem

**Affiliations:** 1 Department of Korean Rehabilitation Medicine, Dongshin Korean Medicine Hospital, 351, Omok-ro, Yangcheon-gu, Seoul 07999, Republic of Korea; sj.joo.kor@gmail.com (S.J.);; 2 Department of Clinical Korean Medicine, Graduate School, Kyung Hee University, 26, Kyungheedae-ro, Dongdaemun-gu, Seoul 02447, Republic of Korea; 3 Department of Acupuncture and Moxibustion, Dongshin Korean Medicine Hospital, 351, Omok-ro, Yangcheon-gu, Seoul 07999, Republic of Korea; 4 Department of Korean Internal Medicine, Dongshin Korean Medicine Hospital, 351, Omok-ro, Yangcheon-gu, Seoul 07999, Republic of Korea; 5 Research Center of Traditional Korean Medicine, College of Korean Medicine, Wonkwang University, 460, Iksan-daero, Iksan 54538, Jeonbuk, Republic of Korea; 6 Hanbang Cardio-Renal Syndrome Research Center, School of Korean Medicine, Wonkwang University, Iksan 54538, Jeonbuk, Republic of Korea

**Keywords:** diabetes mellitus, hyperglycemia, east Asian traditional medicine, integrative medicine, herbal medicine, case report

## Abstract

There is growing interest in alternative therapies for type 2 diabetes mellitus (T2DM) because some patients refuse to receive conventional therapies. In East Asia, herbal medicines are often used to treat T2DM, and modified Gangsimtang (mGST) is prescribed to treat a condition called wasting thirst (消渴), which resembles T2DM. This study reported the treatment of hyperglycemia using herbal medicines without oral hypoglycemic agents or insulin therapy. *Case presentation*: A 36-year-old man with obesity was diagnosed with T2DM four years prior to hospitalization and experienced blood glucose level reduction from 22.2–27.8 mmol/L (400–500 mg/dL) to 5.6–11.1 mmol/L (100–200 mg/dL) by using herbal medicines. He visited D Korean Medicine Hospital with chronic polydipsia and general weakness as chief complaints. He was diagnosed with T2DM on the basis of a hemoglobin A1c level of 11.7% and 2 h postprandial blood glucose level of >25.0 mmol/L (450 mg/dL). Moreover, he was diagnosed with a “dual deficiency of qi and yin” (氣陰兩虛) because of ordinary symptoms (素證). During his 30-day inpatient treatment, the patient received mGST 120 mL thrice daily; as a result, his postprandial blood glucose level decreased from 25.3 mmol/L (455 mg/dL) to 8.6 mmol/L (154 mg/dL), polydipsia decreased (visual analog scale score decreased from six to one), and triglyceride levels decreased from 11.7 mmol/L (1031 mg/dL) to 2.0 mmol/L (174 mg/dL). Plasma glucose levels remained stable for 6 months after the treatment, and no adverse events were observed over 200 days. We administered an herbal decoction to decrease plasma glucose levels without using oral hypoglycemic agents or insulin. *Conclusions*: Herbal decoctions such as mGST can reduce hyperglycemia in patients with T2DM who refuse conventional therapy.

## 1. Introduction

Type 2 diabetes mellitus (T2DM) is characterized by hyperglycemia due to insulin resistance, comprises 90–95% of all diabetes cases [1], and manifests clinically as a metabolic syndrome [2]. Globally, the number of adults with T2DM has tripled in the last 20 years from 151 million in 2000 [3] to 537 million in 2021 [4].

Oral hypoglycemic agents are commonly administered for T2DM. Metformin, which is the first-line drug of choice, is known to cause side effects such as lactic acidosis [5] and vitamin B12 deficiency with long-term use [6]. Increasing the dose or administering combination therapy is common when monotherapy fails to achieve the desired effects [7]. Patients sometimes refuse to receive oral hypoglycemic agents or insulin owing to the inconvenience of receiving multiple medications and their various side effects [8]. Therefore, interest in alternative treatments, such as herbal medicines, is increasing [9].

In East Asia, the administration of herbal medications for treating T2DM is widespread and has a long history. Modern diabetes resembles the east Asian traditional medicine pathology of wasting thirst (WT, 消渴) [10]. The term WT was first used in *Huangdi’s Internal Classic* (黃帝內經; fourth to second century BC), which is the oldest medical text in East Asia that discusses human anatomy, physiology, pathology, diagnosis, treatment, and prevention. The *Synopsis of Prescriptions of the Golden Chamber* (金匱要略; third century) first defined WT as a disease characterized by polydipsia, polyuria, and polyphagia, which are similar to the symptoms of the hyperglycemic hyperosmolar state of T2DM [11], and devoted a separate chapter to the treatment of WT. A combination of Western and east Asian traditional medicine has been widely used in clinical practice to improve blood glucose levels and relieve symptoms in patients with diabetes, with substantial clinical evidence [12,13,14,15,16].

The east Asian traditional medicine treatment is based on syndrome differentiation (辨證) according to the individual complaints of patients. Stomach heat syndrome (胃火熾盛證) and dual deficiency of qi and yin (DQY, 氣陰兩虛) are the most common conditions associated with T2DM [17]. DQY is characterized by dry mouth, dry throat, tired spirit, thirst, poor appetite, spontaneous perspiration, thin body, reddish tongue with less fur, and weak pulse. It is diagnosed when a patient shows three or more of the aforementioned symptoms [17]. Gangsimtang (GST) is an herbal decoction that tonifies DQY and is a prescription recorded in the *Treasured Mirror of Eastern Medicine* (東醫寶鑑) (1613) [18], which was written during the Joseon Dynasty and states the following: “Treats the desire to drink stemming from agitation (煩渴), daily consumption of qi-blood (氣血) due to a heart fire (心火) that flames upward, and impaired kidney water (腎水)”. Trichosanthis Radix (TR) [19], Liriope Platyphylla [20], and Ginseng Radix [21], which are the constituent herbs of GST, have been used to manage blood sugar and reduce related symptoms in patients with diabetes.

Although studies and clinical trials on the use of herbal medicine for treating diabetes have been conducted, research is lacking on the therapeutic effects of herbal medicine without the use of oral hypoglycemic agents or injections in patients with severe hyperglycemia (postprandial plasma glucose level > 25.0 mmol/L). Furthermore, no clinical study has reported the hypoglycemic effect of GST on patients with T2DM. In the current study, we presented a case of a patient who refused conventional medications and was administered GST with dietary restrictions. The treatment resolved hyperglycemic symptoms, reduced blood glucose levels without oral hypoglycemic agents or insulin injections, and maintained optimal plasma glucose levels during a long-term telephone follow-up period.

## 2. Case Presentation

### 2.1. Patient History

We present a case of T2DM in a 36-year-old man with a height of 176 cm, weight of 84 kg, and body mass index (BMI) of 27.1 kg/m^2^ who worked in an office and had low levels of physical activity and irregular eating habits. In 2018, the patient was diagnosed with T2DM at K Korean Medicine Hospital on the basis of a blood glucose level of 22.2–27.8 mmol/L (400–500 mg/dL) and symptoms of general weakness, polydipsia, and polyuria. He was hospitalized for 3 weeks and treated with herbal medicines and acupuncture. Owing to the fear of having to receive oral anti-diabetic medication for the rest of his life, he only received herbal medicine and acupuncture. After inpatient treatment, his blood sugar level was successfully maintained within the range of 5.6–11.1 mmol/L (100–200 mg/dL), leading to his discharge from the hospital with alleviated symptoms. He did not modify his lifestyle or receive medications or insulin treatment for hyperglycemia in the subsequent four years.

In January 2022, the patient was diagnosed with coronavirus disease 2019 and received only symptomatic treatment. In the same year, he was diagnosed with gastroesophageal reflux disease and colon polyps, underwent polypectomy, and received oral medications. When he visited D Korean Medicine Hospital in April 2022, he did not complain of any respiratory or digestive symptoms related to his past medical history. In March 2022, he developed cervical pain caused by prolonged working hours in front of a computer. In April 2022 (10 days before Day 1 of hospitalization), the cervical pain worsened; thus, he underwent acupuncture, cupping, and moxibustion treatment at D Korean Medicine Hospital. During his hospitalization period, the patient received traditional Korean medicine twice daily (acupuncture, moxibustion, and cupping) and extracorporeal shock wave treatment.

### 2.2. Diagnostic Approach in Conventional and East Asian Traditional Medicine

In April 2022 (Day 1), the patient was admitted to D Korean Medicine Hospital with polydipsia and general weakness as chief complaints. On Day 1, diabetes was diagnosed on the basis of a blood test that revealed a plasma glucose level of 20.9 mmol/L (377 mg/dL) and a hemoglobin A1c (HbA1c) level of 11.7%, as measured using the certified method of the National Glycohemoglobin Standardization Program (NGSP). In 2013, the prevalence of type 1 diabetes mellitus (T1DM) in Korea was 46.66 in 100,000 individuals, and the incidence of T1DM in East Asia is among the lowest worldwide [22,23]. The patient had no history of autoimmune diseases, including Hashimoto’s thyroiditis, Graves’ disease, celiac disease, or T1DM. In addition, there was no catabolic presentation, such as unintentional weight loss or ketonuria, and no evidence or history of diabetic ketoacidosis (DKA). Therefore, T1DM was excluded in this patient. Drug-induced diabetes was ruled out because the patient had not received any medications, including glucocorticoids, within the past 1 month. Diseases of the exocrine pancreas were excluded because no other digestive diseases, such as cystic fibrosis or pancreatitis, were diagnosed at the time of polypectomy for colon polyp at the gastroenterology department in January 2022. Moreover, given that there was no family history of diabetes, monogenic diabetes syndrome was excluded.

The patient was diagnosed with T2DM at a different hospital in 2018 owing to insulin resistance because he had obesity with a BMI score of 27.1 kg/m^2^, a triglyceride level of 11.7 mmol/L (1031 mg/dL), and hypertension (≥130/85 mmHg) on Day 1, which implied that the patient had a metabolic syndrome according to the criteria of the National Cholesterol Education Program Adult Treatment Panel III. He was hospitalized for 30 days from April 2022 (Day 1) to May 2022 (Day 30) and was treated using an herbal decoction and dietary restrictions.

The patient complained of frequent fatigue, dry mouth, dry throat, and thirst. Additionally, he complained of spontaneous perspiration. On examination, he had a reddish tongue with less fur. Six of the eight symptoms, including reddish tongue and weak pulse, were present, thus leading to the diagnosis of DQY (Table 1).

### 2.3. Dietary Restriction

During hospitalization, the patient received approximately 1200 calories daily. The patient did not usually have dietary restrictions and consumed a variety of high-calorie snacks. He was allowed to eat one vegetable per day, such as a cucumber or a tomato, to relieve hunger because of dietary restrictions, but no other snacks were allowed.

### 2.4. Herbal Decoction Therapy

Syndrome differentiation (pattern identification) according to east Asian traditional medicine theory led to a diagnosis of DQY (氣陰兩虛). The patient complained of insomnia and elevated blood glucose levels. Therefore, GST was prescribed for DQY and insomnia [18]. GST contains TR (天花粉), recognized as a sovereign medicine (君藥) and a key ingredient in herbal decoction [24] and known for its hypoglycemic effects [19,25]. Liriope Platyphylla (麥門冬) in the mixture has tonifying yin action, while Ginseng Radix (人蔘) has tonifying yin and qi actions. These herbs were administered as part of the modified GST (mGST), which was increased by 50% in the prescription from 8 to 12 g daily, brewed to 360 mL daily, and administered thrice daily starting from Day 4 (120 mL for each administration).

After administering mGST, the 2 h postprandial plasma glucose test showed a slight decrease from 25.0 mmol/L (450 mg/dL) on Day 1 to 17.8 mmol/L (320 mg/dL) on Day 8; thus, TR was increased by 50% from 16 to 24 g/day to further reduce the blood glucose level. To reduce polydipsia, Peurariae Radix (葛根) [14], which acts as an engender fluid (生津) and is commonly used for diabetes mellitus treatment, was prescribed in addition to 8 g of mGST daily and administered in the same dosage from Day 9 (Day 6 of mGST) (Table S1). The patient adhered to the intervention schedule and tolerated the herbal decoction.

## 3. Results

### 3.1. Changes in the Objective Test Results and Subjective Symptoms (Figure 1)

As a result of treatment, the 2 h postprandial plasma glucose levels decreased from 25.3 mmol/L (455 mg/dL) on Day 1 to 8.5 mmol/L (154 mg/dL) on Day 29, and the fasting plasma glucose levels decreased from 15.0 mmol/L (270 mg/dL) on Day 2 to 7.8 mmol/L (141 mg/dL) on Day 30. (Table 2) We performed five blood tests at a 7-day interval for 30 days, with blood drawn in a fasting state after a 10 h fast. HbA1c was measured using an NGSP-certified method, which revealed that HbA1c decreased from 11.7% on Day 1 to 10.7% on Day 21; this result represented a 1.0% decrease over 20 days. The triglyceride level was 11.7 mmol/L (1035 mg/dL) on Day 1 but stabilized between 1.7 and 2.4 mmol/L (149–210 mg/dL) from the second blood test on Day 8 to the fifth blood test on Day 28. Urinalysis showed glucose 4+ and ketones 2+ on Day 1, which improved to glucose (−) and ketones (+/−) on Day 28 (Table 3).

**Figure 1:**
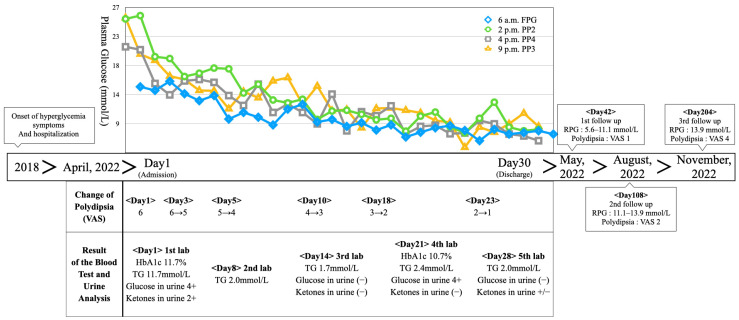
Timeline of the case. Abbreviations: FPG—fasting plasma glucose; PP2—2 h postprandial plasma glucose; PP3—3 h postprandial plasma glucose; PP4—4 h postprandial plasma glucose; VAS—visual analog scale; TG—triglyceride; HbA1c—hemoglobin A1c; RPG—random plasma glucose.

Polydipsia was assessed using a visual analog scale (VAS), which showed a score of six on Day 1. The patient complained of continuous thirst even after occasionally drinking water. The patient drank approximately 4 L of water daily, excluding meals. During his hospitalization, his self-reported thirst decreased along with his plasma glucose level; this led to a decrease in the VAS score to four on Day 5. Furthermore, his drinking volume decreased to 2 L. The patient’s symptoms continued to decrease, with a VAS score of one on Day 23, which was described as barely noticeable. The VAS score remained at one until discharge on Day 30.

**Figure 2:**
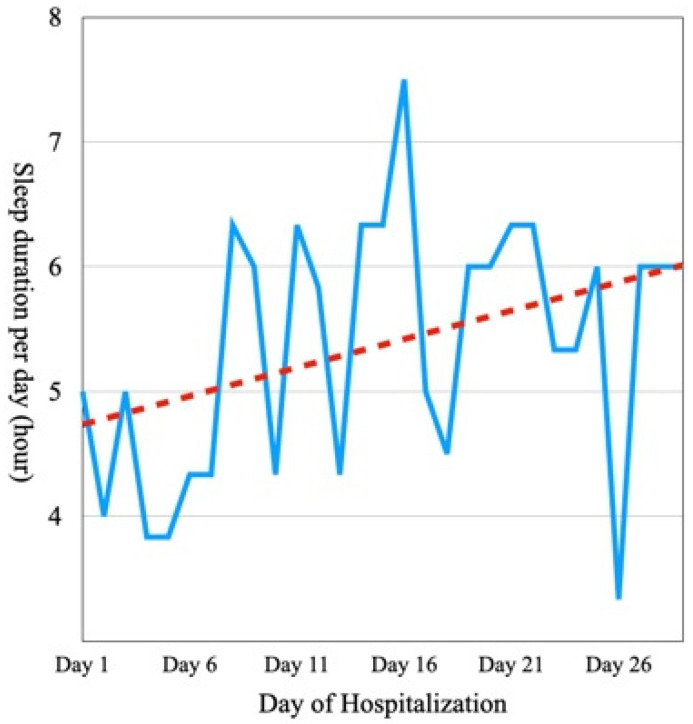
Sleep duration per day during hospitalization. The dotted line is the trend line obtained by linear regression of the sleep duration per day.

From Days 1 to 3, before receiving mGST, the patient slept for an average of 4.7 h per night and complained of insomnia with lethargy and frequent awakenings. However, from Days 27 to 29, he slept for an average of 6 h per night with no interruptions (Figure 2).

After discharge on Day 30, the patient did not receive herbal or acupuncture treatment. The patient visited D Korean Medicine Hospital on Day 42, which was 12 days after discharge. A self-measured plasma glucose test at home showed a distribution of 5.6–11.1 mmol/L (100–200 mg/dL). Associated symptoms, such as polydipsia and polyuria, remained unremarkable. On Day 108, the patient was followed up with via telephone. It was reported that he could not control his diet because of his corporate lifestyle. However, the amount of food he ate was similar to that during hospitalization (approximately half of his normal diet), and his plasma glucose level was maintained at 11.1–13.9 mmol/L (200–250 mg/dL) according to a random plasma glucose test. The polydipsia symptoms had increased slightly since the time of discharge. However, he reported a VAS score of two, which did not significantly interfere with his daily activities. The urinary frequency was maintained at 10 times daily, including 1–2 episodes of nocturia. No other side effects, such as hypoglycemia or weight gain, were observed. On Day 204, the patient was monitored via telephone. It was reported that he was consuming approximately half of his normal diet, and his plasma glucose level was randomly tested two to three times a week and was maintained at approximately 13.9 mmol/L (250 mg/dL). He reported an increase in polydipsia (VAS score of four); however, this did not significantly interfere with his daily life.

The gastrointestinal symptoms commonly associated with herbal medicines, such as heartburn, diarrhea, and nausea, were not reported until the Day 204 follow-up, spanning the entire treatment duration. Blood tests, which were conducted weekly during the hospitalization, included assessments of complete blood count, electrolyte levels, liver function (as indicated by aspartate transaminase, alanine transaminase, and γ-glutamyl transferase), and kidney function (evaluated through blood urea nitrogen and blood creatinine levels). All test results fell within the reference ranges and exhibited no abnormalities, indicating the absence of adverse effects, such as drug-induced liver injury or drug-induced kidney injury.

### 3.2. Patient Perspective

Table 4 shows the patient’s perspective on controlling blood sugar and hyperglycemic symptoms with herbal decoctions only on the basis of the patient’s willingness to forego conventional medication.

## 4. Discussion

### 4.1. Summary of Findings

We presented a case of a patient who was prescribed oral medication after being diagnosed with T2DM but preferred to use herbal decoctions only. He was hospitalized in a Korean hospital for 30 days, during which herbal decoctions and dietary management reduced his blood sugar levels and alleviated his symptoms. To our knowledge, only one case report has been conducted [26] on glycemic lowering and symptomatic improvement using herbal medicine alone in patients with severe diabetes mellitus; symptoms of hyperglycemia, HbA1c ≥ 10%, and plasma glucose level ≥ 16.7 mmol/L (300 mg/dL); and requiring early insulin treatment [7]. In the future, herbal decoctions may be considered for patients with severe diabetes who do not respond to conventional treatments.

### 4.2. Suggested Mechanism of Hypoglycemic Effect

A previous animal study [27] reported that GST extracts significantly decreased plasma glucose levels in hyperglycemic rats compared with the control group. TR, which is the sovereign medicine (君藥) in GST that activates phosphatase on insulin receptors, is the most commonly administered herbal medication for diabetes in Taiwan [19]. Animal studies have identified proteins with hypoglycemic effects in TR [19] and have shown that TR improves renal function in a dose-dependent manner in streptozotocin-induced renal impairment [25]. TR is also used to treat and restore renal function impairment in patients with diabetes. Pueraria lobata is an herbal medication that has been used in East Asia for thousands of years for diabetes treatment and blood sugar level control of patients with T2DM [14,28]. Liriope Platyphylla regulates lipogenesis and lipid uptake in animal studies [29], stimulates insulin secretion from pancreatic beta cells, and reduces abdominal fat deposition [20]. Ginseng Radix reduces the levels of fasting plasma glucose, total cholesterol, interleukin-6, and homeostatic model assessment of insulin resistance [21]. In addition, ginsenoside extracted from ginseng has been used as an adjuvant for patients with diabetes mellitus [30]. The various biologically active constituents of Astragali Radix can protect islet beta cells, reduce plasma glucose, and inhibit insulin resistance [15,31]. Although the diabetic effects of each herbal medication constituting GST are known, data on the diabetic effects of GST, which is a complex herbal formula, have been lacking in previous studies. In the current study, we demonstrated the effect of GST on decreasing blood glucose levels and reducing hyperglycemic symptoms.

Personalized east Asian traditional medicine is one important contributor to this hypoglycemic effect. The diagnosis is based on a comprehensive evaluation of ordinary symptoms (素證) such as food intake and digestion, sleep, and fatigue and symptoms such as tongue conditions and pulse characteristics. DQY causes dry mouth, dry throat, tired spirit, thirst, poor appetite, spontaneous perspiration, weight loss, reddish tongue, less fur, and weak pulse [17,32]. Our patient was initially diagnosed with yin deficiency and heat exuberance (陰虛熱盛) and was prescribed Galgeungeumryeontang (葛根黃芩黃連湯, Gegen Qinlian decoction). However, after observation, this medicine was deemed to be more appropriate for DQY; thus, the prescription was changed to mGST on Day 4 of hospitalization. TR was increased to manage hyperglycemia. The patient had symptoms of reddish tongue, weak pulse, fatigue, and spontaneous perspiration; was diagnosed with DQY; and was prescribed mGST, which is a tonifying yin and qi formula with TR as the sovereign medicine (君藥).

### 4.3. Strengths and Limitations

This study has several limitations. First, it was a single case report; therefore, the effect of mGST requires further study. Second, measurements of serum ketone levels, bicarbonate concentration, blood pH, autoantibody tests, or C-peptide were not conducted. Consequently, the exclusion diagnosis of the T1DM or ketoacidosis exclusion relied solely on clinical evaluation. Third, the hypoglycemic effect was interpreted as a synergistic effect of the herbal decoction and dietary control and was not due to the herbal decoction alone. According to a systematic review and meta-analysis of weight-loss interventions [33], the Mediterranean-style diet study group [34] had the highest HbA1c level reduction of 1.2% over 12 months. By contrast, other diets showed a reduction of ≤0.6%. Therefore, the glycemic lowering effect in this study, including a 1.0% reduction in HbA1c levels after 20 days of hospitalization, is thought to be caused by the synergistic effect of the herbal decoction and dietary control.

Acupuncture could have an effect on reducing blood glucose. However, according to the meta-analysis about acupuncture for T2DM [35], the major acupoints for treating T2DM include ST 36, LI 11, SP 6, BL 20, and LI 4. The patient in this case report had acupuncture therapy only for his cervicalgia. Thus, we used acupoint in his neck muscles such as the scalene, trapezius, and levator scapulae muscles, not acupoint for treating internal diseases like T2DM. Therefore, the hypoglycemic effect in this case was likely caused by the herbal medicine rather than the acupuncture treatment.

This case report has several strengths. First, the patient was under dietary control during hospitalization and was continuously monitored using blood glucose tests four times a day and weekly blood tests. Second, the prescription was personalized by a specialist in traditional Korean medicine with >10 years of clinical experience. Third, although previous studies on the blood sugar–lowering effects of herbs such as TR, Liriope Platyphylla, Ginseng Radix, and Peurariae Radix have been conducted, no study has reported on GST, except for some animal studies [27]. Therefore, the current study is the first case report on GST.

Finally, in a previous case study [26], herbal medicine alone was administered to manage severe diabetes with an HbA1c level of >10%. However, that study was conducted within 1 month from the initial diagnosis of T2DM, and it was impossible to determine the efficacy of a specific herbal prescription during the administration of multiple herbal medications during the treatment period. In the current study, the patient was diagnosed with T2DM more than four years ago and probably had relatively high insulin resistance. Notably, unlike previous studies, this study used a single mGST prescription. In our knowledge, this is the first case report on GST that identified the detailed glycemic changes during hospitalization. In addition, this study explored the therapeutic window of mGST by confirming that no adverse events occurred for 200 days post-treatment. The self-measured plasma glucose tests after discharge showed that blood glucose was maintained to some extent after herbal medicine discontinuation. No dietary restrictions were imposed via telephone follow-up.

### 4.4. Implications for Clinical Practice and Further Study

Given that GST is a prescription recorded in the *Treasured Mirror of Eastern Medicine* (東醫寶鑑) (1613) [18], it is a commonly used prescription in Korea but not in China, Taiwan, or Japan. Therefore, few clinical reports have been conducted on GST for diabetes management, and future clinical studies are required. Case series or prospective observational studies are crucial to investigate the long-term effects of herbal medicine on weight control and diabetes remission, as reported in a previous study that reported diabetes remission via weight control [36]. Because the patient in this case was treated with a combination of dietary restriction, acupuncture treatment, and herbal medicine, it is difficult to reliably distinguish the effects of each intervention in this study. Therefore, further studies are needed to confirm the effects of each intervention and the synergetic effects of these interventions.

Because T2DM is a progressive disease, it is possible for patients to fail to respond to conventional treatment [7]. As this study indicated the effectiveness of herbal medicine in a patient who refused conventional therapy, it is recommended to confirm its effectiveness in patients who do not respond to conventional therapy in the future.

HbA1c tests should be performed at 12-week intervals [37]. Even though the tests were performed only 3 weeks apart in this case, a significant decrease was achieved. Future studies should include HbA1c measurements at intervals of 12 weeks or more to confirm continuous hypoglycemic effects.

DKA stands out as a critical complication of diabetes [38]. In this case, the patient underwent urine ketone measurement but lacked assessment of serum ketone levels, bicarbonate concentration, or pH, which resulted in an incomplete screening for DKA. However, given the patient’s stable vital signs and the absence of clinical symptoms indicative of DKA, the likelihood of DKA is low. As with type 1 diabetes [39], it is worth exploring herbal medicine treatments for DKA in type 2 diabetes through further research.

### 4.5. Suggested Algorithm for Treating Patients Refusing Conventional Therapy

Even though, there are alternative treatments for diabetes mellitus that can be used for patients who refuse conventional therapy. However, in severe cases of diabetes, it is important to use conventional therapy, including insulin injections, to prevent critical complications such as DKA [38]. Given the substantial evidence supporting conventional therapy for patients with T2DM, it is crucial to prioritize the use of interventions such as psychological intervention [40] to encourage patients to undergo conventional therapy, thus preventing critical complications. In the case of patients who persistently refuse conventional therapy, hypoglycemic effects can be achieved through treatments such as herbal medicines with continuous monitoring of blood glucose and vital signs [41]. Above all, strict dietary management and other lifestyle changes are imperative and should be prioritized [35,42].

## 5. Conclusions

In this case report, a patient with severe T2DM, hyperglycemia, and hypertriglyceridemia with hyperglycemic symptoms, such as polydipsia and polyuria, who refused conventional medication was successfully treated with mGST based on TR to decrease plasma glucose levels. After being discharged from the hospital, the patient’s plasma glucose level remained stable via lifestyle management without herbal medicine for 6 months, thus indicating that the effect of the mGST treatment lasted for approximately 6 months; no side effects were observed. For patients with T2DM who refuse conventional treatment, mGST can be considered for glycemic control.

## Figures and Tables

**Table 1:** Symptoms and diagnostic criteria of the dual deficiency of qi and yin (氣陰兩虛). A dual deficiency of qi and yin is diagnosed when a patient has three or more symptoms with a reddish tongue and weak pulse.

Symptoms	Symptoms in This Case	Symptoms	Symptoms in This Case
Dry mouth	(+)	Poor appetite	(−)
Dry throat	(+)	Spontaneous perspiration	(+)
Tired spirit	(+)	Thin body	(−)
Thirsty	(+)	Less tongue fur	(+)

**Table 2:** Change in Plasma Glucose.

(mmol/L)	6 a.m. (Fasting Plasma Glucose)	2 p.m. (2 h Postprandial Plasma Glucose)	4 p.m. (4 h Postprandial Plasma Glucose)	9 p.m. (3 h Postprandial Plasma Glucose)
Day 1		25.3 ***	21.0 **	25.4 ***
Day 2	15.0 *	25.8 ***	20.6 **	19.9 **
Day 3	14.4 *	19.5 **	15.5 *	19.0 **
Day 4	15.8 *	19.3 **	13.8 *	16.6 *
Day 5	13.9 *	16.5 *	15.9 *	16.1 *
Day 6	12.9 *	17.0 **	16.1 *	14.4 *
Day 7	13.7 *	17.8 **	15.7 *	14.4 *
Day 8	10.1	17.7 **	13.7 *	11.7 *
Day 9	11.1 *	14.0 *	12.2 *	14.3 *
Day 10	10.4	15.4 *	15.4 *	13.3 *
Day 11	9.2	13.0 *	11.1 *	15.9 *
Day 12	11.6 *	12.5 *	12.4 *	16.4 *
Day 13	12.3 *	13.1 *	11.1 *	12.3 *
Day 14	9.7	10.0	9.4	15.1 *
Day 15	10.0	11.3 *	13.9 *	11.4 *
Day 16	9.0	11.4 *	8.3	11.5 *
Day 17	9.5	10.8	11.2 *	8.8
Day 18	8.4	10.0	10.6	11.7 *
Day 19	9.2	10.2	12.1 *	11.8 *
Day 20	7.4	8.3	7.8	11.4 *
Day 21	8.1	10.5	9.0	11.0
Day 22	8.8	11.2 *	9.2	9.9
Day 23	9.1	8.9	7.9	9.6
Day 24	8.4	8.0	8.0	5.8
Day 25	6.8	10.2	9.9	8.9
Day 26	8.5	12.7 *	9.4	8.1
Day 27	7.8	8.9	7.8	9.4
Day 28	8.0	8.3	7.5	11.0
Day 29	8.3	8.5	6.8	9.0
Day 30	7.8			

*** >22.2 mmol/L [400 mg/dL], ** 16.7–22.1 mmol/L [300–399 mg/dL], and * 11.1–22.0 mmol/L [200–299 mg/dL].

**Table 3:** Results of the blood test and urine analysis. Blood samples were taken after 10 h of fasting. HDL: high-density lipoprotein.

	Reference Value	Day 1	Day 8	Day 14	Day 21	Day 28
HbA1c in %	4.0–5.6	11.7 *			10.7 *	
Plasma glucose in mmol/L (mg/dL)	3.9–6.1 (70–110)	20.9 * (377) *	18.7 * (336) *	10.8 * (194) *	9.3 * (167) *	7.3 * (131) *
Cholesterol total in mmol/L (mg/dL)	3.5–5.7 136–220	6.7 * (260) *	7.4 * (286) *	6.4 * (247) *	6.4 * (245) *	6.1 * (234) *
Triglyceride in mmol/L (mg/dL)	0.1–1.7 (10–149)	11.7 * (1031) *	2.0 * (176) *	1.7 (149)	2.4 * (210) *	2.0 * (174) *
Glucose in urine	-	4+ *	Non-excution	-	4+ *	-
Ketones in urine	-	2+ *	Non-excution	-	-	+/− *

* out-of-reference value.

**Table 4:** Patient perspective.

Date	Situation	Patient’s Perspective
A day before Day 1	In an outpatient visit for counseling to manage plasma glucose and reduce the symptoms of polydipsia and polyuria.	“I have heard that once you start taking conventional drugs, you have to take them for the rest of your life, so I want to avoid them as much as possible. I have also heard that they have a lot of side effects, and I have heard that even if I take them, my plasma glucose will not be well controlled, so I am reluctant to take them. I have heard that herbal medicines have fewer side effects, so I want to be treated with herbal medicines”.
Day 2	After a blood test on Day 1	“I thought my levels (of blood glucose, triglycerides, etc.) would be bad, but they are worse than I thought. In 2018, my levels were this bad, but I was treated at a traditional Korean medicine clinic and got better. So I think I will get better with traditional Korean medicine this time”.
Day 10	Polydipsia symptoms decreased from a VAS of six (Day 1) to a VAS of three.	“My mouth feels half as dry as it did on my first day in the hospital. I used to go to the bathroom every 3 h, but now it is more like every 4–5 h. I eat less than I used to so I am hungry, but it is manageable. I think I can keep it under control, and I am relieved that my plasma glucose levels continue to improve”.
Day 28	After the discharge decision after the fifth blood test.	“My symptoms of dry mouth and frequent urination have almost disappeared, and I am sleeping better at night and waking up less often than I did at the beginning of my hospitalization. I am glad that my blood glucose levels have improved without conventional medication and that my symptoms have improved so much. I will continue to test my plasma glucose as you taught me after I leave the hospital. I am grateful that my overall condition has improved so much without any side effects during my hospitalization”.
August 2022 (Day 108)	Follow-up over the telephone	“It is not as easy to manage my diet and lifestyle as it was when I was hospitalized because I am living a social life, but when I check my blood glucose often, my blood glucose level is lower than before I was hospitalized (around 11.1–13.9 mmol/L). My symptoms of dry mouth and frequent urination have remained improved, so it’s much easier for me to go about my daily life”.
November 2022 (Day 204)	Follow-up over the telephone	“I have been out of the hospital for about six months now, and my symptoms have not gotten much worse in my daily life. I try, but I am too busy and frustrated to keep up with my diet, but I am still in better condition than I was before I was hospitalized. My blood glucose seems to be a little high, but if it gets higher or my symptoms get worse, I will go back to the doctor for herbal medicine”.

## Data Availability

Data for this case report are stored at D Korean Medicine Hospital and can be accessed via the corresponding author if necessary under relevant laws, including the Personal Information Protection Act and the Medical Service Act of the Republic of Korea.

## Supplementary Materials

The following supporting information can be downloaded at: https://www.mdpi.com/article/10.3390/medicina59111919/s1, Table S1: Prescription of modified Gangsimtang.
