# Supplementary material for: Hypoglycemic Effect of an Herbal Decoction (Modified Gangsimtang) in a Patient with Severe Type 2 Diabetes Mellitus Refusing Oral Anti-Diabetic Medication: A Case Report
Source: Medicina (Kaunas). 2023 Oct 30;59(11):1919. doi: 10.3390/medicina59111919 (PMC10672827; doi:10.3390/medicina59111919)
Supplement: Supplementary file 1 [file medicina-59-01919-s001.zip › medicina-2605691-supplementary.pdf]

Table S1. Prescription of modified Gangsimtang.

Herbal name (in Chinese characters)	Herbal name in Latin	Scientific name of the origin plant	Starting amount (g) per day at Day 4	Changed amount (g) per day at Day 9
Tianhuafen (天花粉)	Trichosanthis Radix	*Trichosanthes kirilowii* Maximowicz	16	24
Maimendong (麥門冬)	Liriope Platyphylla	*Liriope platyphylla* Wang et Tang	12	12
RenCan (人蔘)	Ginseng Radix	*Panax ginseng C. A. Meyer*	12	12
Yuanzhi (遠志)	Polygalae Radix	*Polygala tenuifolia* Willdenow	8	8
Danggui (當歸)	Angelicae Gigantis Radix	*Angelica gigas* Nakai	8	8
Shudihuang (熟地黃)	Rehmanniae Radix Preparata	*Rehmannia glutinosa* Liboschitz ex Steudel	8	8
Fuling(茯苓)	Poria Sclerotium	*Poria cocos* Wolf	8	8
Wuweizi(五味子)	Schisandrae Fructus	*Schisandra chinensis* Baillon	8	8
Gancao(甘草)	Glycyrrhizae Radix et Rhizoma	*Glycyrrhiza uralensis* Fischer	8	8
Huangqi(黃芪)	Astragali Radix	*Astragalus membranaceus* Bunge	8	8
Gegen(葛根)	Peurariae Radix	*Pueraria lobata* Ohwi	(-)	8
Dazao(大棗)	Zizyphi Fructus	*Zizyphus jujuba* Miller var. inermis Rehde	6	6
